# Inhibition of Grb14, a negative modulator of insulin signaling, improves glucose homeostasis without causing cardiac dysfunction

**DOI:** 10.1038/s41598-020-60290-1

**Published:** 2020-02-25

**Authors:** Xunshan Ding, Rugmani Iyer, Christopher Novotny, Daniel Metzger, Heather H. Zhou, Gordon I. Smith, Mihoko Yoshino, Jun Yoshino, Samuel Klein, Gayathri Swaminath, Saswata Talukdar, Yingjiang Zhou

**Affiliations:** 1 Merck & Co., Inc., South San Francisco, CA USA; 2 Merck & Co., Inc., Kenilworth, NJ USA; 3 Center for Human Nutrition, Washington University School of Medicine, St. Louis, Missouri USA

**Keywords:** Diabetes, Medical research

## Abstract

Insulin resistance increases patients’ risk of developing type 2 diabetes (T2D), non-alcoholic steatohepatitis (NASH) and a host of other comorbidities including cardiovascular disease and cancer. At the molecular level, insulin exerts its function through the insulin receptor (IR), a transmembrane receptor tyrosine kinase. Data from human genetic studies have shown that Grb14 functions as a negative modulator of IR activity, and the germline Grb14-knockout (KO) mice have improved insulin signaling in liver and skeletal muscle. Here, we show that Grb14 knockdown in liver, white adipose tissues, and heart with an AAV-shRNA (Grb14-shRNA) improves glucose homeostasis in diet-induced obese (DIO) mice. A previous report has shown that germline deletion of Grb14 in mice results in cardiac hypertrophy and impaired systolic function, which could severely limit the therapeutic potential of targeting Grb14. In this report, we demonstrate that there are no significant changes in cardiac function as measured by echocardiography in the Grb14-knockdown mice fed a high-fat diet for a period of four months. While additional studies are needed to further confirm the efficacy and to de-risk potential negative cardiac effects in preclinical models, our data support the therapeutic strategy of inhibiting Grb14 to treat diabetes and related conditions.

## Introduction

The peptide hormone insulin is produced in the pancreatic beta cells. It is the primary anabolic hormone in the body which regulates the metabolism of carbohydrates, lipids and proteins. It promotes the absorption of glucose into liver, fat and muscle cells. Insulin resistance occurs when the cells fail to efficiently absorb glucose in response to insulin stimulation. It underlies T2D, NASH and a host of other comorbidities including cardiovascular disease and cancer^1^.

Grb14 is a member of the growth factor receptor-bound protein family^2^. It is an intracellular adaptor protein that contains multiple domains including a proline-rich region, a Ras-associating (RA) domain, a pleckstrin homology (PH) domain, a between the PH and SH2 (BPS) domain, and a Src homology 2 (SH2) domain. Grb14 binds to receptors and non-receptor tyrosine kinases and regulates a variety of signaling pathways^2^. Multiple genome wide association studies have found that loci near *Grb14* are significantly associated with waist to hip ratio, blood lipids, body fat percentage and fasting insulin levels in humans, indicating that Grb14 plays a role in regulating metabolism^3–7^.

Grb14 is primarily expressed in metabolically active tissues including liver, fat and skeletal muscle^8,9^. It has been reported that Grb14 binds to the insulin receptor (IR) and negatively regulates the catalytic activity of IR^10,11^. A crystal structure shows that Grb14 binds to IR in a pseudo-substrate inhibitor fashion^11^. The physiological relevance of the regulation of IR by Grb14 was confirmed *in vivo* in the Grb14-KO mice^8^. The whole-body Grb14 deficient mice exhibited improved glucose homeostasis and enhanced insulin signaling. However, the mice exhibited cardiac hypertrophy and impaired cardiac function as measured by echocardiography^12^. This finding raises safety concerns on inhibiting Grb14 as a therapeutic strategy to treat insulin resistance and its related diseases. In this study, we have examined the effects of Grb14 knockdown on metabolism and cardiac function in diet-induced obese (DIO) mice with an AAV carrying shRNA against Grb14 (Grb14-shRNA). We show that Grb14-shRNA results in efficient knockdown of Grb14 in the liver, the white adipose tissue and the heart, and that it improves glucose homeostasis without causing adverse effects on cardiac function in DIO mice.

## Results

### Grb14 expression is negatively correlated with metabolic health in humans

**Figure 1:**
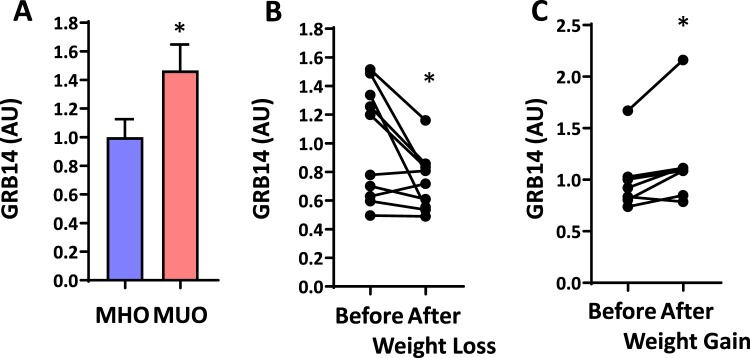
Grb14 Gene Expression in Adipose Tissue Are Negatively Correlated with Metabolic Health in Human. (**A**) Study on metabolically healthy obese (MHO) and metabolically unhealthy obese people (MUO) (n = 15). (**B**) A longitudinal analysis of women with obesity before and after 20 ± 1% weight loss, induced by lifestyle therapy (low-calorie diet) or Roux-en-Y gastric bypass surgery (n = 10). (**C**) A longitudinal analysis of people with MUO before and after 5.9 ± 0.3% weight gain, induced by consuming an additional 1000 kcal/day until a targeted 5–7% weight gain was achieved (n = 7). *P < 0.05, Student’s t-test.

It was previously reported that Grb14 regulates insulin signaling^2,8,10^. To determine if this regulation is relevant in humans, we analyzed the expression of the *GRB14* gene in human adipose tissues (Table 1). The RNA-seq analysis showed that Grb14 expression was significantly higher in metabolically unhealthy obese (MUO) human subjects compared to metabolically healthy obese (MHO) subjects (Fig. 1A). In addition, the expression of *GRB14* in adipose tissues was decreased significantly in women with obesity after weight loss through either lifestyle intervention or bariatric surgery (Fig. 1B). Furthermore, the Grb14 expression in adipose tissues was increased significantly in MUO subjects after weight gain induced by consumption of extra calories (Fig. 1C).

**Table 1:** Characteristics of the clinical study subjects Group 1 (n = 30).

	MHO (n = 15)	MUO (n = 15)
Body mass index (kg/m^2^)	36.8 ± 0.9	38.3 ± 1.1
Fasting glucose (mg/dl)	88 ± 1	97 ± 3*
Fasting insulin (µU/ml)	10.2 ± 0.8	24.9 ± 3.3*
Glucose infusion rate during the HECP (µmol/kg FFM/min)	44.8 ± 3.7	26.7 ± 1.8*
**Group 2 (n = 10)**		
	**Baseline**	**After weight loss**
Body mass index (kg/m^2^)	39.7 ± 1.8	31.7 ± 1.4^†^
Fasting glucose (mg/dl)	90 ± 2	85 ± 2^†^
Fasting insulin (µU/ml)	19.2 ± 2.3	7.9 ± 0.8^†^
Glucose infusion rate during the HECP (µmol/kg FFM/min)	48.3 ± 6.5	65.2 ± 5.1^†^
**Group 3 (n = 7)**		
	**Baseline**	**After weight gain**
Body mass index (kg/m^2^)	35.4 ± 1.6	37.5 ± 1.7^†^
Fasting glucose (mg/dl)	106 ± 3	111 ± 5
Fasting insulin (µU/ml)	28.3 ± 4.8	32.7 ± 7.3
Glucose infusion rate during the HECP (µmol/kg FFM/min)	29.6 ± 5.4	24.9 ± 4.3^†^

Data are means ± SEM.

MHO = metabolically healthy obese; MUO = metabolically unhealthy obese; HECP = hyperinsulinemic-euglycemic clamp procedure, FFM = fat-free mass.

*Value significantly different from the corresponding value in the MHO group, *P* < 0.05.

^†^Value significantly different from the corresponding Baseline value, *P* < 0.05.

### Grb14 knockdown improved glucose homeostasis

**Figure 2:**
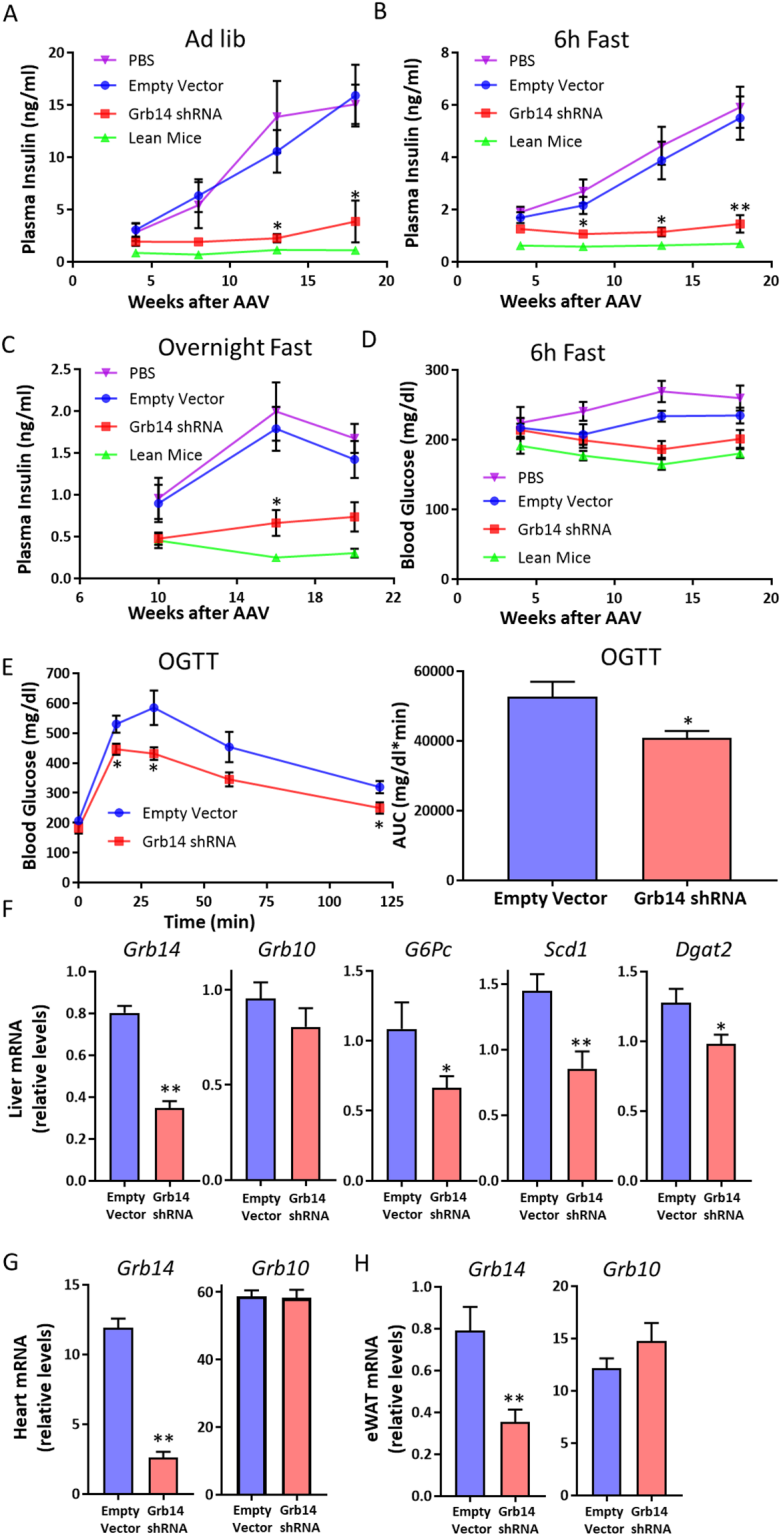
Grb14 Knockdown Improves Glucose Homeostasis. (**A**) Ad lib plasma insulin during an 18-week high-fat diet (HFD) in C57BL/6J male mice (n ≥ 6). (**B**) 6h-fast plasma insulin during an 18-week HFD in C57BL/6J male mice (n ≥ 6). (**C**) Overnight-fast plasma insulin during a 20-week HFD in C57BL/6J male mice (n ≥ 6). (**D**) 6h-fast blood glucose during an 18-week HFD in C57BL/6J male mice (n ≥ 6). (**E**) Oral glucose tolerance test after a 16-week HFD in C57BL/6J male mice (n ≥ 6). AUC: area under curve. (**F**) QPCR analysis of gene expression in the liver after a five-month HFD in C57BL/6J male mice (n ≥ 6). (**G**) QPCR analysis of gene expression in the heart after a five-month HFD in C57BL/6J male mice (n ≥ 6). (**H**) QPCR analysis of gene expression in the epididymal white adipose tissue (eWAT) after a five-month HFD in C57BL/6J male mice (n ≥ 6). *P < 0.05, **P < 0.01 Empty Vector vs Grb14 shRNA, Two-way ANOVA for (**A**–**D**), Student’s t-test for (**E**–**H**).

To investigate the potential effects of pharmacological inhibition of Grb14, we generated an AAV carrying shRNA against Grb14 (Grb14-shRNA) and an AAV carrying the empty vector as control. To determine the knockdown efficiency of the Grb14-shRNA, we injected AAVs to the normal chow-fed mice via tail vein and collected the liver, epididymal white adipose tissue (eWAT), skeletal muscle, and heart four weeks after injection. Grb14 gene expression was decreased by ~90% in the liver and ~80% in the heart and in the white adipose tissue in the mice treated with Grb14-shRNA compared to the empty vector control (Fig. S1). To assess the metabolic effects of Grb14 inhibition, mice were injected with Grb14-shRNA or the empty vector and were challenged with a diet containing 60% calories from fat (high-fat diet, HFD) immediately after the AAV injection. HFD feeding induced hyperinsulinemia in mice and the treatment with Grb14-shRNA markedly decreased plasma insulin levels in mice under various nutritional states compared with the empty vector (Fig. 2A–C). Following 13 weeks of AAV and HFD treatment, the Grb14-shRNA-treated mice also had a trend towards lower plasma glucose levels compared to the PBS- or empty vector-treated control mice after a six-hour fast, although the difference didn’t reach statistical significance in comparison to the empty vector-treated mice (Fig. 2D). In oral glucose tolerance tests, Grb14-shRNA-treated mice had a reduced glucose excursion compared to the empty vector-treated mice (Fig. 2E). No significant change was found in ad lib blood glucose levels, overnight fasting blood glucose levels, body weight or food intake (Figs. S2 and S3).

We terminated the study and collected tissues from mice five months after the AAV treatment. Grb14 knockdown was confirmed with QPCR analysis. Grb14-shRNA treatment resulted in ~60% downregulation, ~80% downregulation and ~50% downregulation of Grb14 gene expression in the liver, heart and white adipose tissue respectively (Fig. 2F–H). We next compared the expression of metabolic genes in the liver of mice treated with Grb14-shRNA or the empty vector for five months. The gluconeogenic gene glucose-6-phosphatase (*G6Pc*) was down-regulated significantly with Grb14-shRNA treatment (Fig. 2F). Stearoyl-Coenzyme A desaturase 1 (*Scd1*) and diacylglycerol O-acyltransferase 2 (*Dgat2*), two lipogenic genes, were also decreased with Grb14-shRNA treatment (Fig. 2F). Expression of other examined genes were not statistically different between the two groups (Figs. 2F and S4).

### Grb14 knockdown did not impair hemodynamic cardiac function

**Figure 3:**
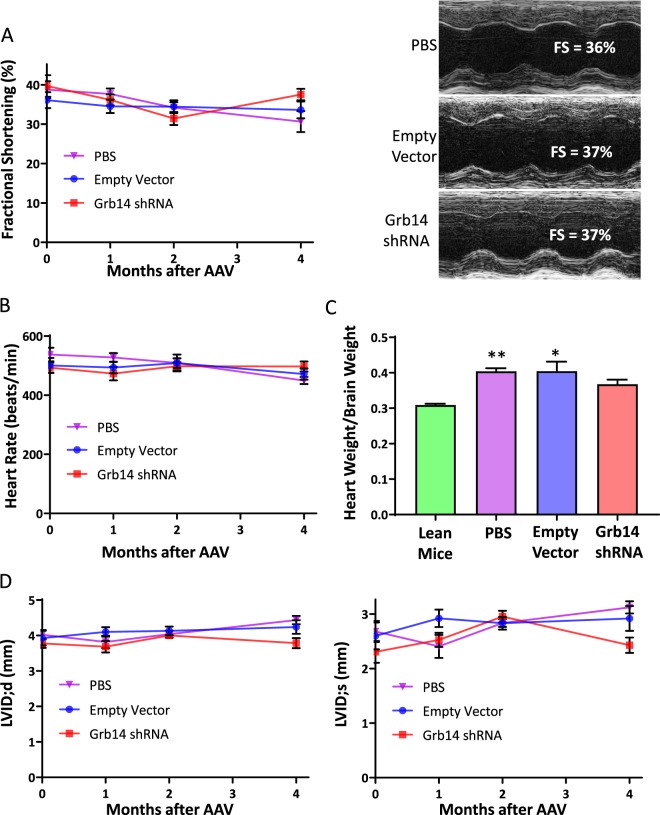
Grb14 Knockdown Did Not Cause Cardiac Dysfunction. (**A**) Fractional shortening (FS) during a four-month HFD in C57BL/6J male mice (n ≥ 6). Shown on the right are representative images of echocardiographic measurements (M-mode) of hearts at four months. (**B**) Heart rate during a four-month HFD in C57BL/6J male mice (n ≥  6). (**C**) Heart weight normalized to brain weight after a five-month HFD in C57BL/6J male mice (n ≥  6). (**D**) Left ventricular internal diameter (LVID) thickness during a four-month HFD in C57BL/6J male mice (n ≥ 6). d: systolic s: diastolic. *P < 0.05, **P < 0.01 Compared to lean mice, One-way ANOVA.

A previous study shows that mice with germline deficiency of Grb14 exhibits cardiac hypertrophy and dysfunction as measured by heart weight and fractional shortening^12^. To assess the potential effects of Grb14 inhibition on cardiac function, we first performed a Grb14 knockdown experiment using siRNA in neonatal rat cardiomyocytes. As shown in Fig. S5, two different Grb14 siRNAs efficiently silenced *GRB14* gene expression. However, only minor impact was observed on the hypertrophic gene signature. To investigate the functional impact of Grb14 knockdown on cardiac function *in vivo*, we performed echocardiography longitudinally in DIO mice treated with the AAV carrying Grb14-shRNA or the empty vector for up to four months. No difference in fractional shortening or heart rate was observed between Grb14-shRNA- and the empty vector-treated mice measured at one, two and four months after the AAV injection (Fig. 3A,B).

In line with the literature^13^, HFD treatment led to cardiac hypertrophy as measured by heart weight normalized to brain weight (Fig. 3C). However, no difference in heart weight was observed between Grb14-shRNA- and the empty vector-treated mice (Fig. 3C). In addition, no difference was observed between Grb14-shRNA- and the empty vector-treated mice in key hemodynamic parameters, including left ventricular internal diameter, inter ventricular septum thickness, left ventricular anterior wall thickness, and left ventricular posterior wall thickness (Figs. 3D and S6).

### Insulin-dependent interaction between Grb14 and IR

**Figure 4:**
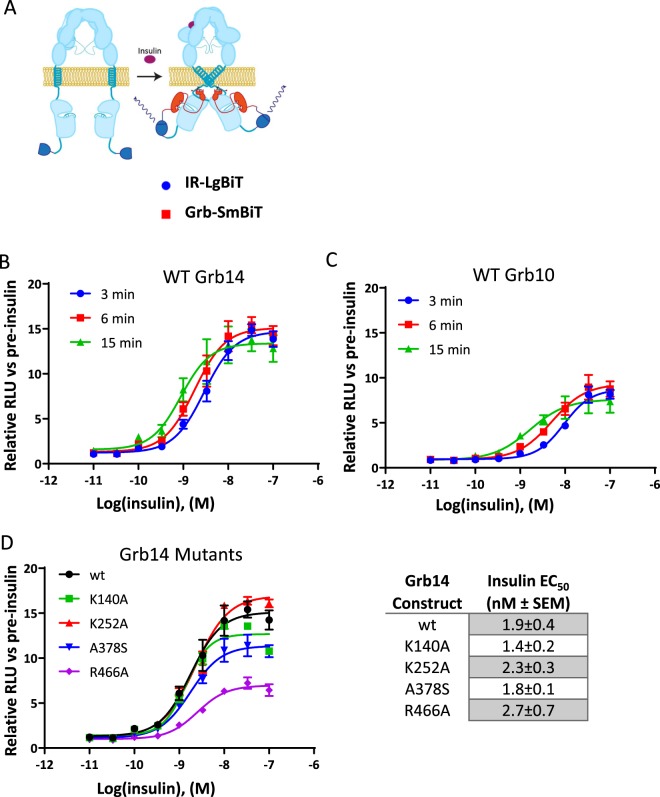
A Split NanoLuc Assay for Grb14-IR Interaction. (**A**) Scheme of the study design. (**B**) Insulin-dependent interaction between Grb14 and IR at the indicated time point (n = 2). (**C**) Insulin-dependent interaction between Grb10 and IR at the indicated time point (n = 2). (**D**) The effect of varying concentrations of insulin on the interaction between wild-type (wt) or the indicated Grb14 mutant was plotted 6 minutes after insulin addition. The calculated EC_50_ of insulin for each construct is shown to the right (n = 2).

In order to screen for Grb14 inhibitors, we established a split Nanoluciferase assay where the LgBiT portion of the enzyme was fused to the C-terminus of the insulin receptor and the SmBiT tag was fused to the C-terminus of Grb14^14^ (Fig. 4A). We confirmed the insulin-dependent interaction between Grb14 and IR, which showed a time -dependent change of EC_50_ from 2.9 nM at 3 min to 0.9 nM at 15 min post insulin addition (Figs. 4B and S7A). We also established a split Nanoluciferase assay based on the interaction between Grb10 and IR as a method to assess the selectivity of compounds. Like Grb14, the IR-Grb10 interaction was dependent on insulin stimulation with an EC50 of 9.4 nM at 3 min and 1.7 nM at 15 min post insulin addition (Figs. 4C and S7B).

In order to evaluate the relative contribution of each domain within Grb14 to the interaction between IR and Grb14, we introduced point mutations in different domains previously reported to abrogate their ability to bind to the respective binding partners^11,15^. While none of the mutations altered the EC_50_ of insulin for the stimulation of the IR-Grb14 interaction, the R466A mutant showed a reduced maximum luciferase signal compared to the wild-type Grb14 (Fig. 4D). However, we were unable to rule out the possibility that this mutation only altered the proper assembly of the Nanoluciferase enzyme rather than diminished the inhibition of the IR signaling.

## Discussion

It is estimated that over one third of adults in the US have insulin resistance^16^. However, thiazolidinediones (TZDs) remain the only class of FDA-approved insulin sensitizers. The undesired side effects associated with the use of TZDs include weight gain, edema and bone fracture^17^. Therefore, there is a large unmet medical need for effective and safe insulin sensitizers.

Multiple studies have shown that Grb14 negatively regulates the insulin signaling. Mice with the germline global KO of Grb14 show improvement in glucose homeostasis and in the insulin signaling in liver and skeletal muscle. In humans, Grb14 gene expression decreases following gastric bypass surgery and increases in obese patients in skeletal muscle^18^. Furthermore, our data demonstrate that there is a negative correlation between Grb14 gene expression in adipose tissue and metabolic health in humans (Fig. 1), even though in the current study gene expression was only analyzed in adipose tissues in humans and therefore is unable to capture changes in liver and skeletal muscle. Despite the potential benefits of inhibiting Grb14 in disease settings, one study has shown that the global Grb14 KO mice have cardiac hypertrophy accompanied by impaired cardiac function, raising the potential safety concern for developing therapies of inhibiting Grb14 activity. Using an AAV carrying an shRNA against Grb14, we show here that efficient knockdown of Grb14 in the liver, the adipose tissue and the heart significantly improved glucose homeostasis without inducing cardiac hypertrophy or dysfunction. There are several possible reasons why Grb14 inhibition did not cause cardiac dysfunction in our study. First, in contrast to germline KO mice that do not express Grb14 from embryo, in our study the deficiency of Grb14 was introduced in adulthood. Grb14 deficiency during embryonic and fetus development possibly leads to cardiac dysfunction. Second, it is also possible that the deletion of the *GRB14* genes in tissues beyond the heart may contribute to the cardiac dysfunction in the Grb14 global KO mice. Moreover, the complete deficiency of Grb14 through genetic engineering in the prior study may result in cardiac dysfunction, compared with the partial deficiency of Grb14 by shRNA knockdown in the current study. Finally, the previous study was performed on lean mice while the current study was done under an HFD, which by itself causes cardiac hypertrophy and dysfunction^13,19^. Nonetheless, our data demonstrate the separation between the beneficial effects on metabolism and the undesired cardiac dysfunction with Grb14 inhibition. Although further studies are needed to better understand the role of Grb14 in the cardiovascular system and the translatability of the animal data for humans, our study suggests the feasibility of inhibiting Grb14 to treat insulin resistance.

Protein-protein interaction (PPI) is critical in signal transduction pathways. The pharmaceutical industry has been pursuing modulators that either enhance or disrupt PPI for drug development for decades^20^. Despite the tremendous efforts invested, limited success has been achieved. Disruption of PPI remains challenging in drug discovery, especially for intracellular targets as in the case of Grb14. However, multiple features make Grb14 a promising candidate for this approach. First, RA, PH, BPS and SH2 domains in Grb14 are all critical for the inhibition of IR^2^. In addition, serine phosphorylation in the BPS domain interferes with the interaction with IR^21^, offering another potential point for manipulation. Finally, a recent report has successfully identified a small molecule that disrupts the binding of Grb14 to IR and enhances insulin signaling in cellular systems^22^, demonstrating the potential of this approach in drug discovery. In addition to PPI inhibition, Grb14 is also a candidate suitable for other pharmaceutical modalities such as proteolysis-targeting chimera (PROTAC), anti-sense oligonucleotide (ASO) or siRNA.

The studies presented here focus on the role of Grb14 in glucose metabolism and cardiac function. Using a similar shRNA approach, an earlier study shows that silencing of Grb14 leads to decreased expression of lipogenic genes and lower triglyceride content in the liver in addition to improved glucose homeostasis in mice^23^. We also found that Grb14-shRNA reduced expression of the lipogenic genes *Scd1* and *Dgat2* in the liver (Fig. 2F). Therefore, it is intriguing to speculate that Grb14 inhibition might also be beneficial in the context of fatty liver disease.

While our data clearly show that knockdown of Grb14 with shRNA is sufficient to improve glucose homeostasis, we cannot pin down the primary tissue accounting for the efficacy due to the lack of tissue selectivity of our approach. It will be interesting to address this question with tissue-specific Grb14-KO mice. The answer to this question should lead to a better understanding of the biology of Grb14 and guide rational design of selective Grb14 inhibitors for therapeutic purpose.

## Materials and Methods

### Clinical studies

A total of 47 men and women participated in this study, as part of their involvement in other studies that were conducted in the Clinical and Translational Research Unit at Washington University School of Medicine (ClinicalTrials.gov, NCT02706262, NCT00981500, NCT01299519, and NCT01184170). All subjects completed a screening medical evaluation that included a history and physical examination, a resting electrocardiogram, and standard blood tests. All studies were approved by the Institutional Review Board of Washington University School of Medicine in St. Louis, MO, and written informed consent was obtained from all subjects before their participation. In addition, all experiments were performed in accordance with relevant guidelines and regulations.

Study 1 was a cross-sectional analysis of participants with metabolically healthy obesity (MHO; n = 15, 40 ± 2 years old, 1 man and 14 women), defined as BMI 30–50 kg/m2 and fasting plasma glucose concentration <100 mg/dl, 2-h OGTT plasma glucose concentration <140 mg/dl, and HbA1c ≤ 5.6%, or metabolically unhealthy obesity (MUO) (n = 15, 42 ± 2 years old, 1 man and 14 women), defined as BMI 30–50 kg/m2 and fasting plasma glucose concentration ≥100 mg/dl, or 2-h OGTT plasma glucose concentration ≥140 mg/dl, or HbA1C ≥ 5.7%. Study 2 was a longitudinal analysis of women with obesity before and after 20 ± 1% weight loss, induced by lifestyle therapy (low-calorie diet) or Roux-en-Y gastric bypass surgery (n = 10, 45 ± 3 years old, BMI = 39.7 ± 1.8 kg/m2). Study 3 was a longitudinal analysis of people with MUO before and after 5.9 ± 0.3% weight gain, induced by consuming an additional 1000 kcal/day until a targeted 5–7% weight gain was achieved (n = 7, 52 ± 3 years old, 4 men and 3 women BMI = 35.4 ± 1.6 kg/m2). The effects of weight loss and weight gain on body composition, insulin sensitivity and expression of other adipose tissue genes have been reported previously^24–26^.

After subjects fasted for 12 h overnight, a hyperinsulinemic-euglycemic clamp procedure was conducted to assess whole-body insulin sensitivity. An intravenous catheter was inserted into a forearm vein to infuse insulin and dextrose and an additional catheter was inserted into a radial artery to obtain arterial blood samples. Insulin was infused at a rate of 50 mU/m^2^ body surface area (BSA)/min (initiated with a two-step priming dose of 200 mU/m^2^ BSA/min for 5 min followed by 100 mU/m^2^ BSA/min for 5 min). Euglycemia (~100 mg/dL) was maintained by variable rate infusion of 20% dextrose. Blood samples were obtained before beginning the hyperinsulinemic-euglycemic clamp procedure to determine basal, postabsorptive plasma glucose and insulin concentrations. Biopsies from abdominal subcutaneous adipose tissue were obtained before starting insulin and dextrose infusion while subjects laid at rest in bed. The periumbilical area was cleaned and anesthetized with 1% lidocaine injection, and a small skin incision (~0.5 cm) was made. Adipose tissue was then aspirated through a 4-mm liposuction cannula (Tulip Medical Products, San Diego, CA) connected to a 60-cc syringe. Samples were immediately rinsed in ice-cold saline and frozen in liquid nitrogen before being stored at −80 °C until further processing.

Plasma glucose concentration was measured by an automated glucose analyzer (Yellow Springs Instruments Co.). Plasma insulin concentration was measured by using electrochemiluminescence technology (Elecsys 2010; Roche Diagnostics). Total RNA was isolated from frozen subcutaneous adipose tissue samples by using QIAzol and RNeasy mini kit (QIAGEN). Library preparation was performed with total RNA and cDNA fragments were sequenced on an Illumina HiSeq-4000. The fragments per kilobase million reads (FPKM) values were calculated and used for further gene expression analyses. The RNA-seq data have been deposited in the NCBI’s Gene Expression Omnibus database (GEO GSE143319).

### Generation of recombinant adenoviral associated viral vector (AAV) expressing shRNA against mouse Grb14

To generate AAV encoding potent short hairpin RNA (shRNA) against mouse Grb 14, we followed a previously described protocol^27^. Briefly, ten short hairpin RNA (sh1-sh10) of 19 nucleotides, with the starting position at nucleotide 536, 437, 294, 321, 1577, 660, 1320, 485, 1211, 1547 (NM_016719) respectively, were designed to specifically target mouse Grb14. Forward and reverse oligonucleotides were annealed and ligated into the BbsI site of AAV cis plasmid containing the human H1 promoter to drive shRNA expression. To screen for an shRNA that efficiently inhibit mouse Grb14, we transfected the ten candidate AAV-shRNAs into Hepa1c1c7 cells that express mouse Grb14 endogenously using 4D-Nucleofector (Lonza, Allendale, NJ) per the manufacturer’s instruction. Cells were harvested 48 hours post transfection, and RNA was isolated and prepared for RT-PCR analysis. The most potent shRNA (sh6) was selected for AAV9 packaging. AAV9-H1-Grb14sh6 and the negative control AAV vector that contains the H1 promoter and termination sequence were produced by the helper-free triple-plasmid transfection method at University of Massachusetts Medical School Viral Vector Core. The AAV cis plasmid, an adenoviral helper plasmid and a chimeric packaging plasmid containing the AAV2 Rep gene and AAV9 Cap gene were co-transfected into HEK293 cells. AAV vectors were subsequently purified by two rounds of cesium chloride density gradient ultracentrifugation, and titers were determined via RT-PCR analysis.

### Mouse experiments

Male C57BL/6J mice were purchased from the Jackson Laboratory. Mice were fed with standard chow or an HFD containing 60% fat-derived calories (Research Diets, D12492) ad libitum. For AAV treatments, mice were randomized based on body weight and ad lib blood glucose levels. 10-week old mice were injected with AAV (9 × 10^11^ viral genome copies/mouse) via tail vein and immediately subjected to the HFD challenge. All experiments were approved by the Institutional Animal Care and Research Advisory Committee of Merck & Co., Inc., South San Francisco, CA, USA and were performed in accordance with relevant guidelines and regulations.

### Oral glucose tolerance test (OGTT)

After 16-h overnight fast, blood glucose was measured using AlphaTrak glucometer via tail bleeding and recorded as baseline values. Mice were then administered with glucose (20 g/100 ml, in saline solution) via oral gavage at the dose of 2 g/kg body weight. Blood glucose was measured via tail bleeding at 15, 30, 60, and 120 minutes post glucose administration.

### Plasma insulin measurements

Blood was collected via tail bleeding into Microvette tubes coated with EDTA (Sarstedt, Germany) and spun at 8,000 rpm for ten minutes in a bench-top centrifuge at 4 °C to separate plasma. Insulin levels were measured using a commercial insulin ELISA kit (Chrystal Chem, IL) according to the manufacturer’s instruction.

### Quantitative real-time PCR (QPCR)

Total RNA was extracted from tissues with RNeasy mini kits (Qiagen). Two micrograms of RNA from each sample were then used to generate cDNA with SuperScript™ VILO™ cDNA Synthesis Kit (Invitrogen). All TaqMan probes were purchased from Life Technologies. QPCR was run on Applied Biosystems ViiA 7 system. The data were analyzed using the delta-delta Ct method by using PPiB as the internal control^28^.

### Echocardiography

Echocardiograms was acquired by short axis M-mode under spontaneous respiration with 1–2% isoflurane in an oxygen mix. Images were acquired with the use of a Vevo 2100™ High-Resolution *In Vivo* Imaging System (Visual Sonics, Toronto, Canada), at heart rates >350 bpm. Electrocardiogram and heart rate were monitored throughout the imaging procedure. Echocardiographic measurements were taken from the two-dimensional short axis (m-mode) recordings of the LV. For each parameter, three images from consecutive cardiac cycles were measured and averaged. One reader analyzed all the echocardiograms in a blinded manner.

### Split nanoluciferase (NanoLuc) assay

The sequence corresponding to the insulin receptor isoform B was Gibson cloned into the LgBiT C-terminal fusion plasmid (Promega cat#N2014), the sequence corresponding to Grb14 was Gibson cloned into the C terminal SmBiT fusion plasmid (Promega cat#N2014) using standard protocols. Construct accuracy was confirmed by Sanger sequencing. HEK293T cells (ATCC cat#CRL-3216) were maintained in DMEM (Gibco cat#11995-065) plus 10% FBS (Gibco cat#26140-079) at 37 °C and 5% CO2.

For the split NanoLuc assay 4,000 HEK 293T cells were plated per well in a TC treated white opaque 96 well plate (Corning cat#3917). The plate was placed in the incubator and the cells were allowed to adhere overnight. The following day the cells were transfected with the InsR-LgBiT and Grb14-SmBiT or in a 2.5:1 ratio using Fugene HD (Promega cat#E2311) according to the manufacturer’s instructions and the plate was returned to the incubator. 24 hours later the media was changed to 100 µL of DMEM without serum and the cells were placed back in the incubator. 16 hours later the media was exchanged for fresh serum starve media. 2 h later the Nano-BiT protocol was run using a dose response of insulin according to the manufacturers protocol. Briefly, the plate was equilibrated to room temperature for 10 min. 25 µL of the NanoBit substrate and buffer (Promega Cat#N2014) is added to each well and the luminescence is read for 5 min on the EnVision (PerkinElmer). At the completion of the 5 min read 10 µL of insulin or PBS diluted in DMEM with no serum to provide the indicated final concentration of insulin was added to each well and luminescence was read every 1.5 minutes for 30 minutes. The luminescence signal after the addition of insulin was normalized to the luminescence signal for that individual well at the end of the 5-minute pre-insulin read.

### Neonatal rat cardiomyocytes experiment

Neonatal rat cardiomyocytes were isolated from the hearts of 14 neonatal SD rats using Cellutron Life Technology (NC 6031) kit following the manufacturer’s instruction. All the solutions (SureCoat, D1, D2 + EC, D3, NS medium) were diluted and prepared per the manufacturer’s protocol. Briefly the 24-well plates were coated with pre-warmed SureCoat solution until further use. The neonatal rats were sterilized with 70% ethanol before and after decapitation. The chest was opened with sharp scissors and the heart was quickly removed and placed in 50 mL falcon tube containing ice-cold D1 buffer. The hearts were washed twice with ice-cold D1 buffer. The buffer was carefully removed, and the hearts were transferred to a 25 mL beaker containing a magnetic stir bar. The digestion buffer (D2 + EC) was added to the beaker and the beaker was placed on a magnetic stirrer at 37 °C (~150 rpm) and incubated for 12 min. The mixture was pipetted a few times after the solution started becoming cloudy due to collagenase activity. The cloudy supernatant was then transferred to a 15-mL falcon tube and centrifuged at 300 × g for 2 min at room temperature. The supernatant was removed and the pellet was resuspended in 5 mL of D3 solution. The procedure from addition of D2 + EC to the tissue to resuspension of pellet containing the cells in D3 solution was repeated until all the tissue was digested by collagenase (4×−5×). All the resuspended solution was collected in a falcon tube and was filtered through cell strainer. Finally, the cells were counted and pre-plated for 45 min in an uncoated plate and incubated at 37 °C, 5% CO2 to reduce contamination from cardiac fibroblasts. After 45 min, the unattached cells were carefully removed and gently washed with 1X NS medium. The cells were then centrifuged at 300 × g and the pellet was resuspended in NS medium and plated at ~1 × 10^6^ cells/well in a 24-well SureCoat-treated plate. Small interference RNA (siRNA) was purchased from Thermo Fisher Scientific and transfection was performed according to the manufacturer’s instruction.

### Statistical analyses

Statistical analyses were conducted with Prism 8.1.1 (GraphPad, San Diego, CA). Comparisons between multiple groups across multiple time points were analyzed using two-way analysis of variance (ANOVA), followed by Turkey’s multiple comparison test. Comparison of heart weight between groups was analyzed using one-way ANOVA. Comparisons between two groups were analyzed using Student’s t-test. Statistical significance was defined as P < 0.05.

## Supplementary information

Supplementary Data.
