# Supplementary material for: Inhibition of Grb14, a negative modulator of insulin signaling, improves glucose homeostasis without causing cardiac dysfunction
Source: Sci Rep. 2020 Feb 25;10:3417. doi: 10.1038/s41598-020-60290-1 (PMC7042267; doi:10.1038/s41598-020-60290-1)

**Supplementary Data**

**Inhibition of Grb14, a negative modulator of insulin signaling, improves glucose homeostasis without causing cardiac dysfunction**

Xunshan Ding#^1^, Rugmani Iyer#^1^, Christopher Novotny#^1^, Daniel Metzger^1^, Heather H Zhou^2^, Gordon I. Smith^3^, Mihoko Yoshino^3^, Jun Yoshino^3^, Samuel Klein^3^, Gayathri Swaminath^1^, Saswata Talukdar^1^, Yingjiang Zhou*^1^

^1^Merck & Co., Inc., South San Francisco, CA, USA

^2^Merck & Co., Inc., Kenilworth, NJ, USA

^3^Center for Human Nutrition, Washington University School of Medicine, St. Louis, Missouri, USA.

#These authors contributed equally to the work

*Correspondence and requests for materials should be addressed to YZ (Yingjiang.Zhou@merck.com)

**SUPPLEMENTARY FIGURE LEGENDS**

Figure S1 Efficient Grb14 Knockdown

QPCR analysis of Grb14 gene expression in the liver, the heart, the epididymal white adipose tissue (eWAT), and the quadriceps muscle four weeks after AAV injection in C57BL male mice (n = 3).

* P< 0.05, ** P< 0.01 Empty Vector vs Grb14 shRNA, Student’s t-test

Figure S2. Effects of Grb14 Knockdown on Ad Lib or Overnight-fast Blood Glucose

(A) Ad lib blood glucose during an 18-week high-fat diet (HFD) in C57BL/6J male mice (n ≥ 6).

(B) Overnight-fast blood glucose during a 20-week HFD in C57BL/6J male mice (n ≥ 6).

Figure S3. Effects of Grb14 Knockdown on Body Weight and Food Intake

(A) Body weight during an 18-week high-fat diet (HFD) in C57BL/6J male mice (n ≥ 6).

(B) Daily food intake during an 18-week HFD in C57BL/6J male mice (n ≥ 6).

Figure S4. Effects of Grb14 Knockdown on Liver Gene Expression

QPCR analysis of gene expression in the liver after a five-month HFD in C57BL/6J male mice (n ≥ 6).

Figure S5. Effects of Grb14 Knockdown on Gene Expression in Neonatal Rat Cardiomyocytes

QPCR analysis of gene expression 48 hours post siRNA transfection. NC siRNA; negative control siRNA.

* P< 0.05, ** P< 0.01 Compared to NC siRNA, Student’s t-test

Figure S6. Grb14 Knockdown Did Not Cause Cardiac Dysfunction

(A) Inter ventricular septum (IVS) thickness during a four-month HFD in C57BL/6J male mice (n ≥ 6).

(B) Left ventricular anterior wall (LVAW) thickness during a four-month HFD in C57BL male mice (n ≥ 6).

(C) Left ventricular posterior wall (LVPW) thickness during a four-month HFD in C57BL male mice (n ≥ 6).

d: systolic s: diastolic

Figure S7. A Split NanoLuc Assay for Grb14-IR Interaction

(A) Insulin-dependent interaction between Grb14 and IR (n=2).

(B) Insulin-dependent interaction between Grb10 and IR (n=2).


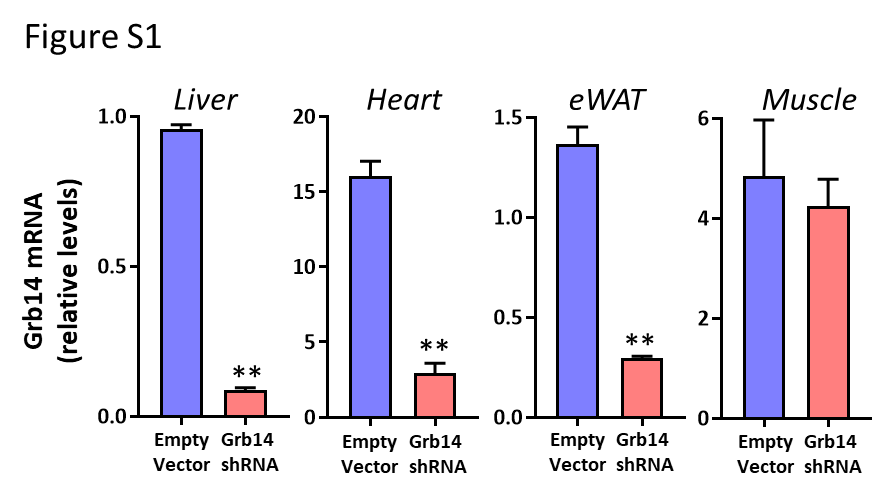


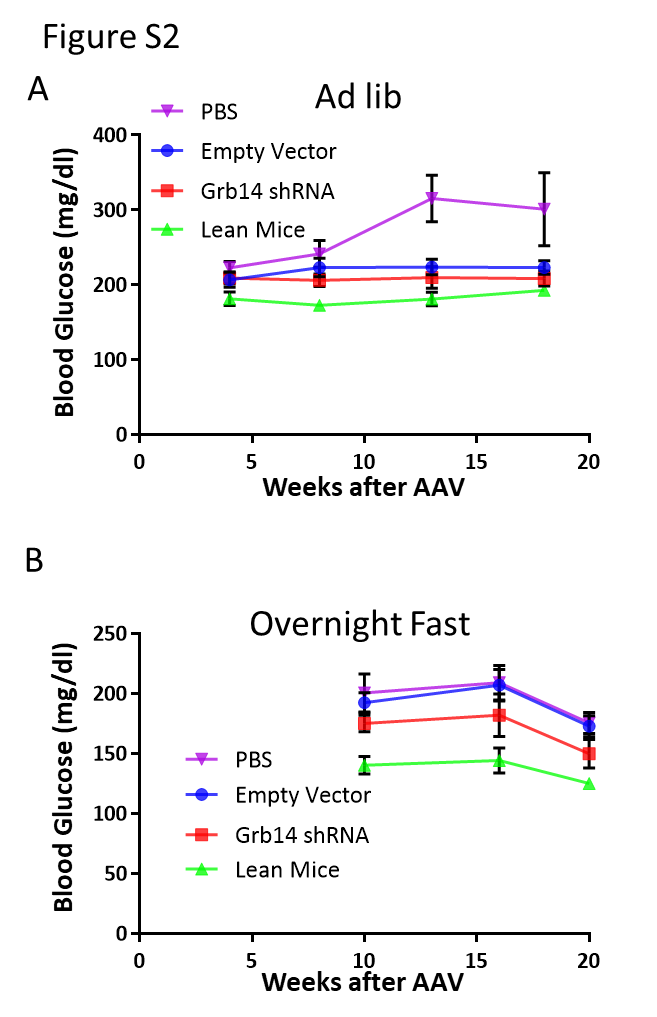


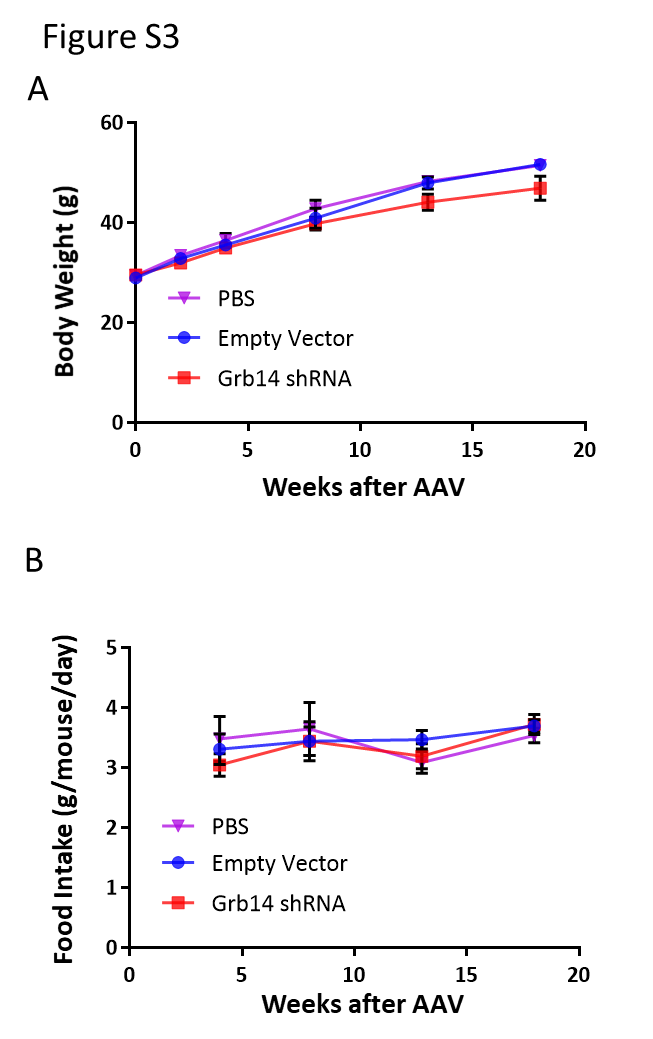


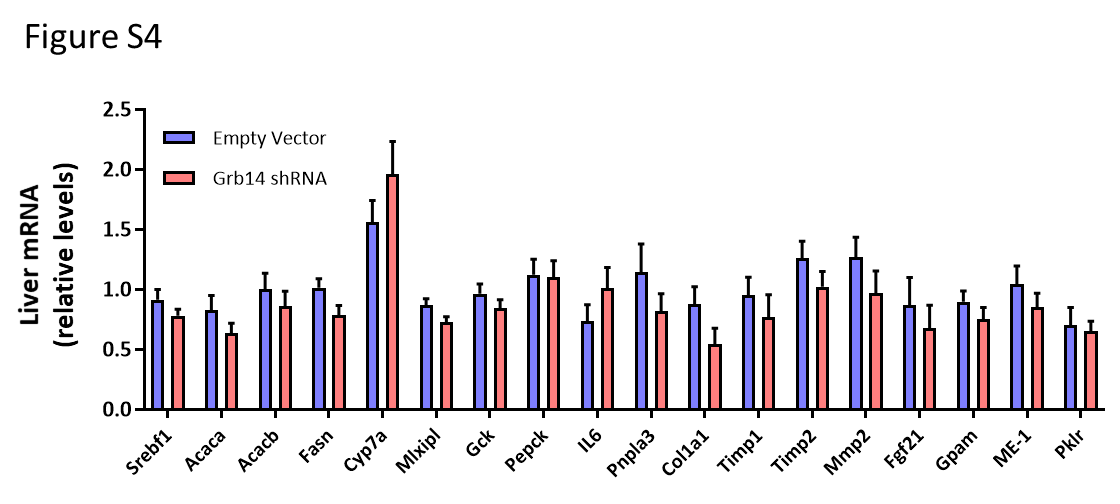


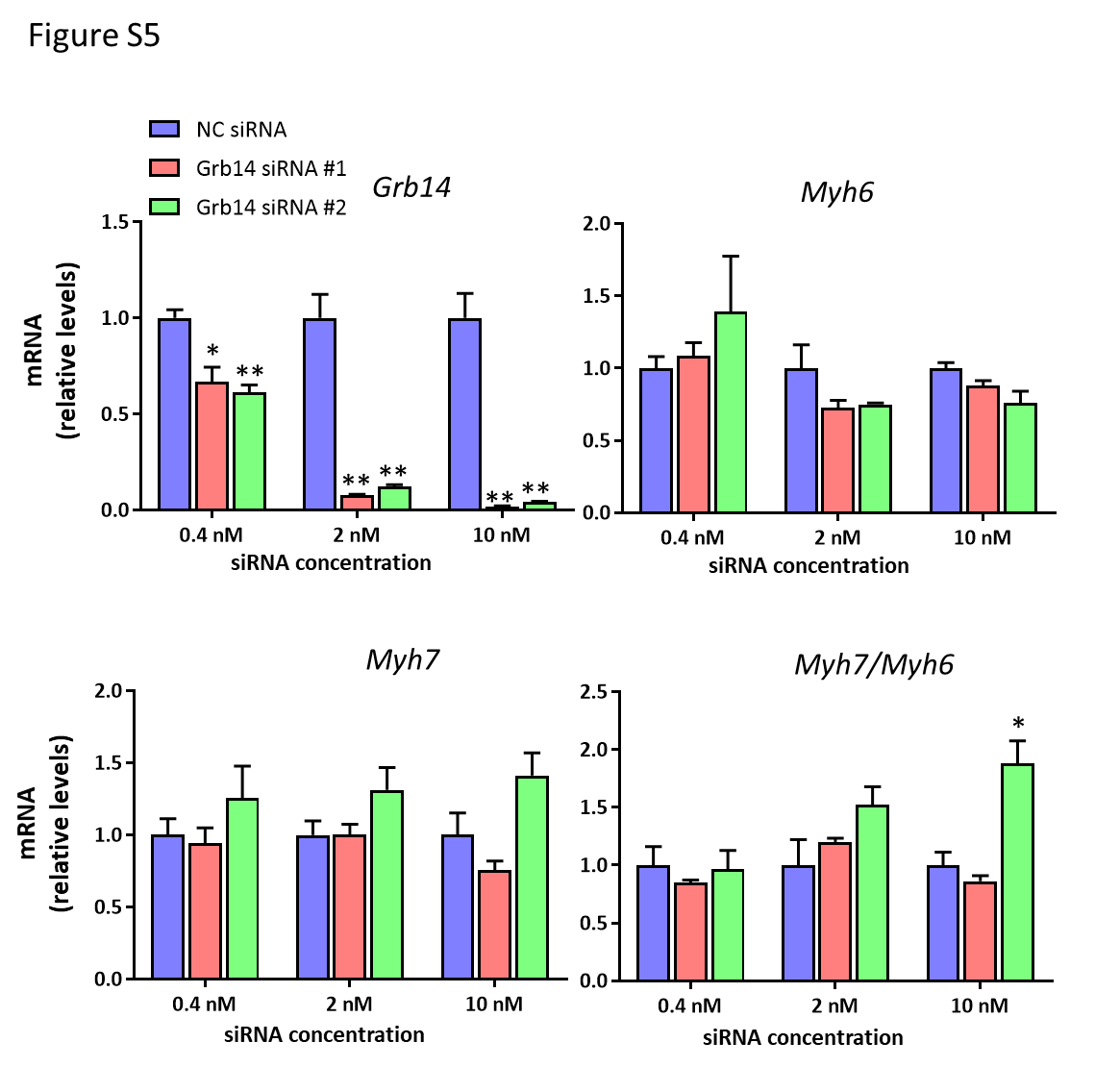


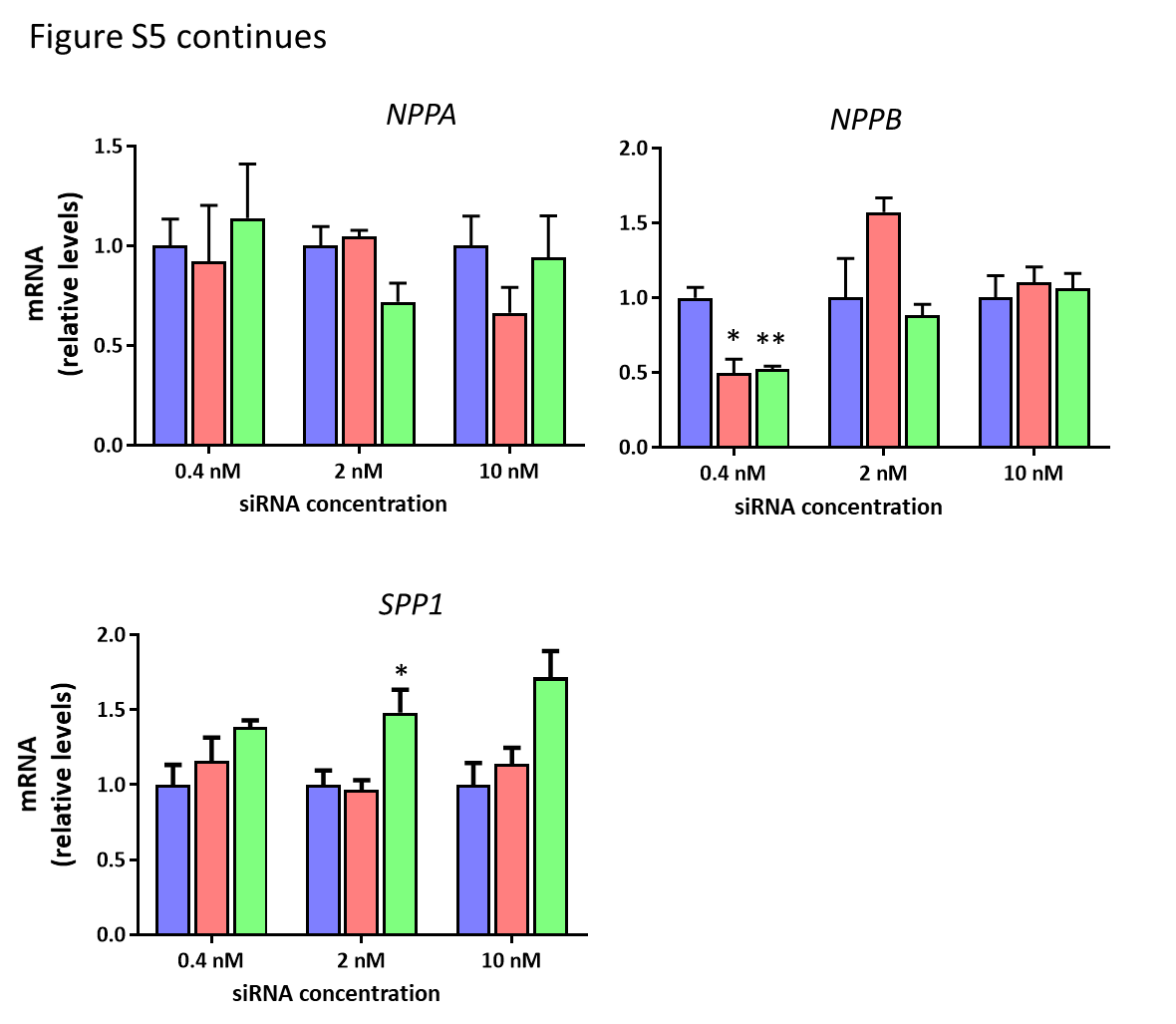


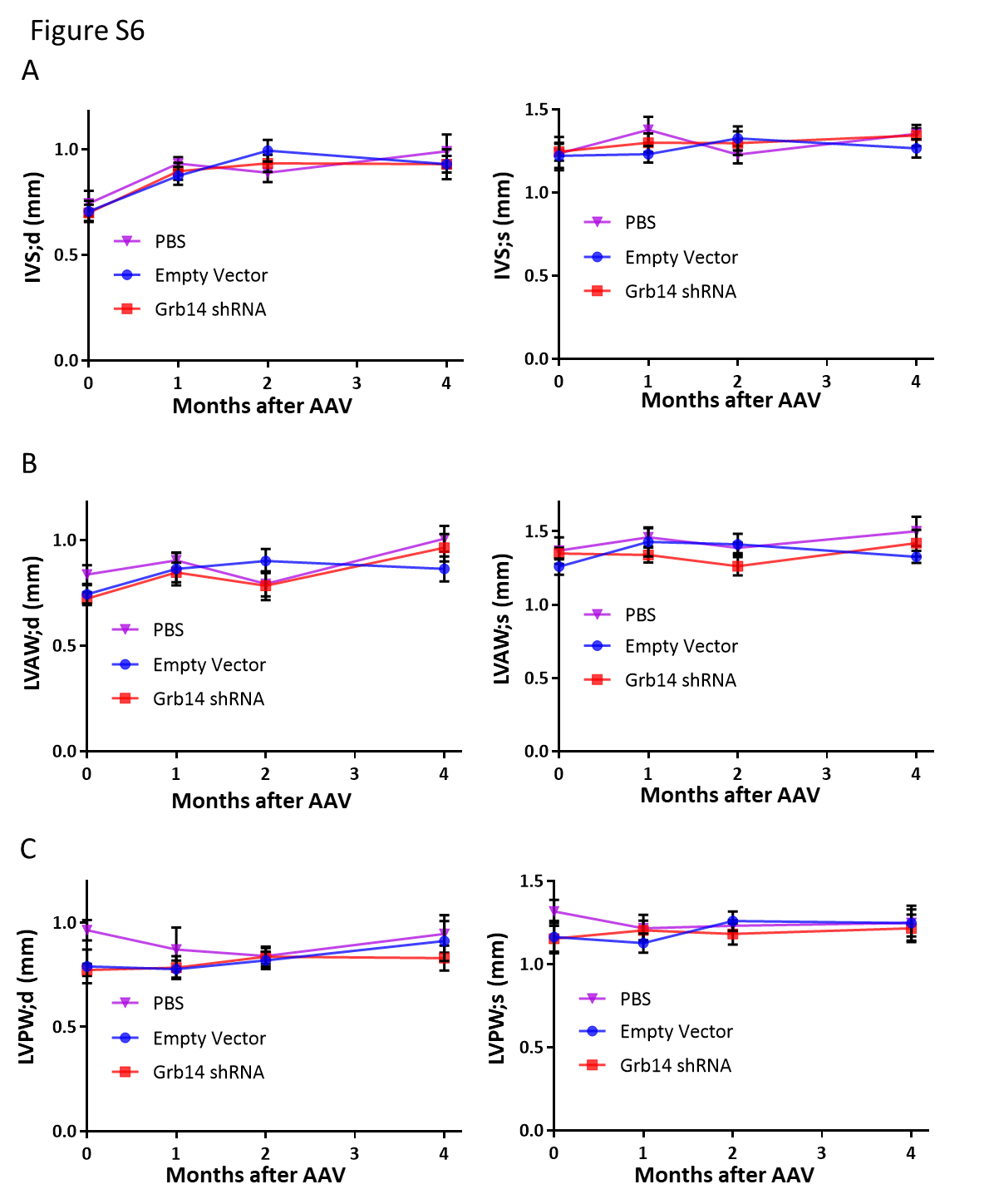


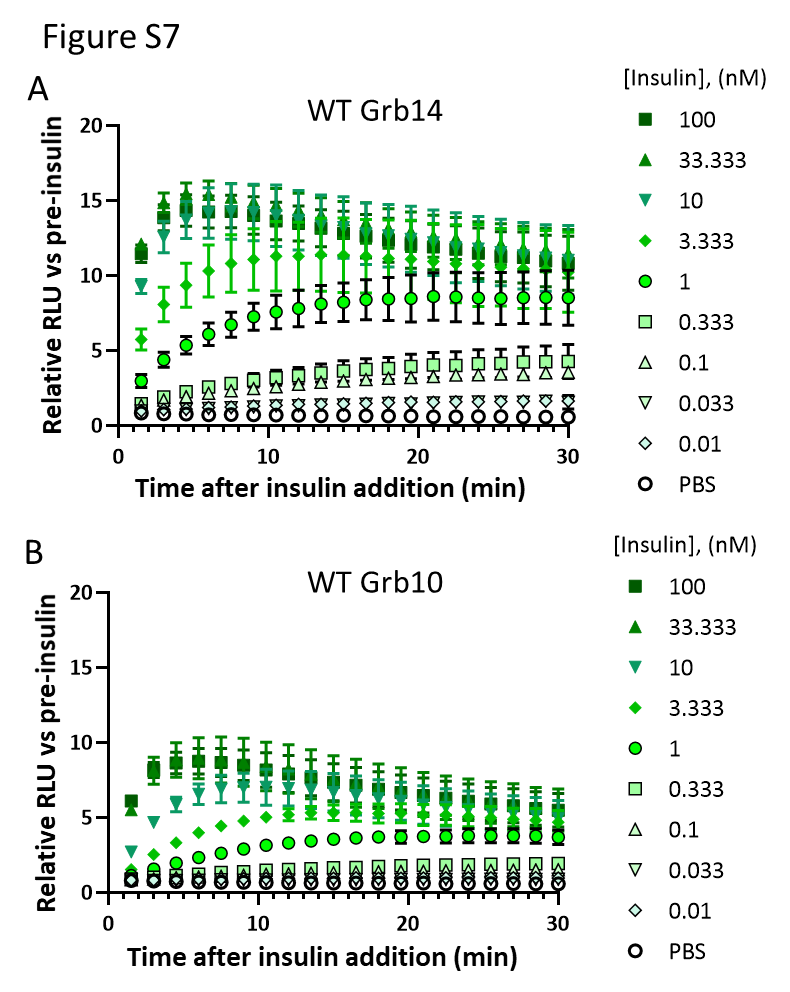

Supplement: Supplementary file 1 — Supplementary Data. [file 41598_2020_60290_MOESM1_ESM.docx]
